# Adrenocortical Carcinoma

**DOI:** 10.3390/cancers13051077

**Published:** 2021-03-03

**Authors:** Alfredo Berruti, Guido Alberto Massimo Tiberio, Sandra Sigala

**Affiliations:** 1 Medical Oncology Unit, Department of Medical and Surgical Specialties, Radiological Sciences and Public Health, University of Brescia, ASST Spedali Civili of Brescia, 25123 Brescia, Italy; alfredo.berruti@gmail.com; 2 Surgical Unit, Department of Medical and Surgical Specialties, Radiological Sciences and Public Health, University of Brescia, ASST Spedali Civili of Brescia, 25123 Brescia, Italy; guido.tiberio@unibs.it; 3 Department of Molecular & Translational Medicine, Section of Pharmacology, University of Brescia, 25123 Brescia, Italy

**Keywords:** adrenocortical carcinoma, biomarkers, drug therapy, surgery, disease management

Adrenocortical carcinoma (ACC) is an extremely rare disease, the incidence of which is 0.7–2 per million population per year in Western countries [1]. The management of this disease is challenging.

This Special Issue comprises 22 papers (18 original articles, 4 reviews) presented by international leaders in the field of ACC. The four review papers focus on topics of great interest. Two of these refer to ACC genomics and provide a complete overview of the potential role of epigenetic alterations [2] and non-coding RNA molecules [3] in the processes of the pathogenesis, diagnosis, and prognostication of ACC. The other two reviews address two important clinical topics. Adjuvant therapy is a relevant issue in the management of ACC. The disease is often aggressive, and post-operative therapy in radically resected patients could improve the cure rate. Mitotane is the standard adjuvant therapy [1,4], but the efficacy of this drug in this setting could be potentially improved by chemotherapy. However, there is a critical need to establish risk calculators to predict rates of recurrence and to implement molecular profiling of ACC to guide adjuvant therapy [5]. In this respect, a Dutch paper published in this Special Issue, reporting the results of the development of a clinical prediction model for ACC-specific mortality, could be of relevance [6]. Another important issue is the large impact of ACC and related therapies on the health-related quality of life (HRQoL) of patients. The fourth review paper underlines the need for HRQoL research in patients with ACC, with the aim of improving patient-centered care [7].

Among the 18 original articles, 7 refer to prognostic parameters and 1 has been previously mentioned. Additionally, another paper assumed the role of investigating the effect of mitotane serum levels on patient prognosis [8]. The premise for this study lies in the fact that studies that have demonstrated the therapeutic value of mitotane concentrations within the so-called therapeutic range (14–20 mg/L) were based on the peak mitotane level and do not provide an adequate representation of chronic exposure to mitotane [9]. This paper demonstrates—for the first time—that the time in target range (TTR), defined as the number of months in which mitotane concentrations are ≥14 mg/L, also has prognostic significance [8]. Four articles report the results of explorative studies on the prognostic role of the counts of two tissue markers: sterol-O-acyl transferase 1 (SOAT1) [10] and CD8+-cytotoxic T lymphocyte [11], the latter being assessed in a pediatric series. Two blood parameters were also assessed in the form of circulating tumor cells [12] and miR-483-5p [13]. Another paper showed that a pervasive circulating autoantibody response against the cancer testis antigen FATE1—which is detected both in pediatric and adult ACC patients—is negatively correlated to disease-free survival (DFS) and overall survival (OS) in adult patients and is associated with decreased immune response-associated gene expression [14]. As a whole, the results presented in these papers are promising and we really hope that at least some of the encouraging data included within them can be confirmed in validation series and, thus, assist in obtaining a greater panel of prognostic factors in the future.

Three papers deal with the genetics of ACC. In one of these, it is shown that the prevalence of altered mismatch repair (MMR) genes among pediatric patients is higher than that in colorectal and endometrial cancer cohorts [15]. The second paper examines germline variants of phosphodiesterase (PDE) enzymes. The results show that PDEs and other regulators of the cAMP signaling pathway may contribute to pediatric adrenocortical tumorigenesis, perhaps by cooperating with germline hypomorphic mutant TP53 alleles and uniparental disomy of chromosome 11p15 (Beckwith–Wiedemann syndrome) [16]. Both of these observations have clinical and translational implications. The third genetic paper explores the association of two single nucleotide polymorphisms (SNPs) CYP2W1*6 and CYP2B6*6 in predicting the individual response to mitotane. The two SNPs analysis demonstrate opposite roles—the CYP2W1*6 SNP is associated with a reduced probability to reach the mitotane therapeutic range, whereas CYP2B6*6 is correlated with higher mitotane levels [17]. 

As regards the three preclinical published studies, one of them explored the effect of crosstalk between cancer cells and adipose precursors in the stroma using a co-culture in vitro model. The results suggest that adipose cell precursors could promote cancer cell reprogramming and invasion, opening new perspectives on the mechanisms of ACC aggressiveness and the development of effective therapeutic approaches [18]. The second study provides—for the first time—a demonstration of the antineoplastic activity of trabectedin, an anti-tumor drug isolated from the Caribbean tunicate *Ecteinascidia turbinata* against ACC cells. In addition, the synergistic cytotoxic activity of trabectedin with mitotane provides the rationale for testing this combination in a clinical study [19]. The third preclinical study uses mouse xenografts bearing human ACC to test the potential synergy between mitotane and radiation therapy. The results show that mitotane could have radiosensitizing properties and, when given in combination with radiotherapy, it is able to increase neoplastic growth inhibition and cell death [20].

Three clinical papers evaluated the efficacy of therapeutic strategies. Etoposide, doxorubicin, and cisplatin plus oral mitotane (EDP-M) [21] represent the reference regimen in the management of patients with adrenocortical carcinoma (ACC) [1,4]. The efficacy of this regimen is limited. However, while we are waiting for new treatment strategies, we have to utilize the EDP-M scheme to its greatest potential. One institution experience showed that early disease progression in EDP-M-treated cases does not indicate treatment inefficacy. In addition, surgery of residual disease in partially responding patients allowed for the detection of pathologically complete responses in a few of them, and Ki67 expression of post-chemotherapy residual disease could be an additional prognostic factor that deserves to be studied further [22]. The disease response to systemic therapy is usually evaluated with the Response Evaluation Criteria In Solid Tumors (RECIST) criteria. A clinical paper published in this Special Issue shows that the assessment of disease response to chemotherapy might be improved by concomitant evaluation of three different criteria, i.e., RECIST, Choi, and volumetric. The results show that the disease response, concordantly assessed by all three criteria, allows for the identification of a patient subset with a more favorable outcome [23]. ACC frequently recurs in the peritoneum after primary surgical resection. Therefore, strategies aiming to prevent peritoneal recurrence could potentially improve patient outcomes. One paper presents the results of a single institution experience regarding the potential efficacy of hypertermic intra-peritoneal chemotherapy (HIPEC) after radical surgery. The results show that this therapeutic procedure might result in long-term disease control, particularly in treatment-naïve patients [24].

Finally, another paper addresses the issue of whether the hypothalamic–pituitary–adrenal (HPA) axis can recover after cessation of adjuvant mitotane therapy for ACC. Data from 23 patients with pathologically proven ACC, treated with adjuvant mitotane for a minimum of two years, were analyzed, and the results showed that a high proportion of patients achieved HPA axis recovery following cessation of mitotane adjuvant therapy [25]. However, it should be noted that complete recovery is often delayed by up to 2.5 years and a regular assessment of the hormonal profile is required.

In conclusion, this Special Issue presents some significant progress made in fields related to the genetic and pathophysiology of ACC and outlines some of the advances in the treatment and prognostication of ACC patients. Above all, this Special Issue highlights how bringing together researchers and clinicians from around the world is key to better understanding this challenging disease and improving the available therapeutic strategies.
